# Sym004-induced EGFR elimination is associated with profound anti-tumor activity in EGFRvIII patient-derived glioblastoma models

**DOI:** 10.1007/s11060-018-2832-6

**Published:** 2018-03-21

**Authors:** Stephen T. Keir, Vidyalakshmi Chandramohan, Carlee D. Hemphill, Michael M. Grandal, Maria Carlsen Melander, Mikkel W. Pedersen, Ivan D. Horak, Michael Kragh, Annick Desjardins, Henry S. Friedman, Darell D. Bigner

**Affiliations:** 1 Preston Robert Tisch Brain Tumor Center at Duke, Department of Neurosurgery, Duke University Medical Center, 3624 DUMC, Baker House, Durham, NC 27710 USA; 2 Symphogen A/S, Pederstrupvej 93, 27500 Ballerup, Denmark

**Keywords:** GBM, Sym004, EGFR, EGFRvIII, mAbs

## Abstract

**Background:**

Sym004 is a mixture of two monoclonal antibodies (mAbs), futuximab and modotuximab, targeting non-overlapping epitopes on the epidermal growth factor receptor (EGFR). Previous studies have shown that Sym004 is more efficient at inducing internalization and degradation of EGFR than individual components, which translates into superior cancer cell inhibition. We investigated whether Sym004 induces removal of EGFRvIII and if this removal translates into tumor growth inhibition in hard-to-treat glioblastomas (GBMs) harboring the mutated, constitutively active EGFR variant III (EGFRvIII).

**Methods:**

To address this question, we tested the effect of Sym004 versus cetuximab in eight patient-derived GBM xenograft models expressing either wild-type EGFR (EGFRwt) and/or mutant EGFRvIII. All models were tested as both subcutaneous and orthotopic intracranial xenograft models.

**Results:**

In vitro studies demonstrated that Sym004 internalized and removed EGFRvIII more efficiently than mAbs, futuximab, modotuximab, and cetuximab. Removal of EGFRvIII by Sym004 translated into significant in vivo anti-tumor activity in all six EGFRvIII xenograft models. Furthermore, the anti-tumor activity of Sym004 in vivo was superior to that of its individual components, futuximab and modotuximab, suggesting a clear synergistic effect of the mAbs in the mixture.

**Conclusion:**

These results demonstrate the broad activity of Sym004 in patient-derived EGFRvIII-expressing GBM xenograft models and provide a clear rationale for clinical evaluation of Sym004 in EGFRvIII-positive adult GBM patients.

**Electronic supplementary material:**

The online version of this article (10.1007/s11060-018-2832-6) contains supplementary material, which is available to authorized users.

## Importance of the study

Glioblastoma (GBM) is an aggressive, highly invasive tumor with a median survival of 12–15 months. GBM tumors are associated with multiple genetic mutations and alterations, with approximately 50% having EGFR gene amplification. Approximately half of GBMs with EGFR gene amplification express a truncated EGFR (EGFRvIII) from a mutated gene lacking exons 2–7, which encode part of the extracellular ligand-binding domain. The truncated receptor displays a constitutive, ligand-independent tyrosine kinase activity and is associated with a more aggressive tumor phenotype than tumors expressing only EGFRwt. Therefore, new therapies active against EGFRvIII, such as Sym004, stand to improve treatment strategies for patients and warrant further exploration.

## Introduction

Glioblastoma (GBM) is the most common, aggressive and subsequently lethal tumor of the central nervous system in adults [1, 2]. Although GBM therapy has improved in recent years, the prognosis for this disease still remains dismal, with median survival rates of 42.4% at 6 months and 17.7% at 1 year [2, 3]. While numerous clinical trials seeking further improvements are underway, the current standard of care still involves maximum safe surgical resection or tumor debulking, followed by concomitant radiation and temozolomide (TMZ) therapy [4–6]. Despite encouraging advances in recent scientific research focusing on the genetic origins and molecular drivers of GBM, only modest improvements have been made in treating this fatal disease [7]. The poor prognosis associated with GBM results largely from a lack of understanding of its aggressive nature and insufficient therapeutic options [8]. Thus, there is a clear need for new treatments and targeted therapies for patients diagnosed with GBM [3, 9, 10].

Data from The Cancer Genome Atlas (TCGA)—National Cancer Institute project recently proposed various subtypes of GBM, where each subtype is characterized by its own distinct set of molecular properties and genetic aberrations [11]. TCGA data indicate that approximately 67% of all patients diagnosed with GBM have an aberrant amplification, deletion, or mutation of one or more receptor tyrosine kinase(s) [12]. The most frequent genetic change associated with GBM is amplification of the EGFR gene, which results in overexpression of the transmembrane tyrosine kinase receptor and occurs in approximately 57% of the GBM patient population [12, 13]. In 50% of these cases, the EGFR amplification is accompanied by a gene rearrangement known as EGFRvIII [11, 13–15]. EGFRvIII is caused by an in-frame deletion of 801 base pairs of the coding sequence from exons 2–7, resulting in a truncated gene encoding an aberrant receptor lacking two-thirds of the extracellular domain [16, 17]. EGFRvIII is unable to bind EGFR ligands, but the receptor is constitutively active.

The over-expression of EGFR and/or its constitutively activated variant, EGFRvIII, is a major characteristic of GBM and is associated with aggressive, invasive, and therapeutic-resistant phenotypes [8]. The GBM cell line U87MG, which was retrovirally transfected with EGFRvIII (U87MG.EGFRvIII), showed significant growth advantage when grown as tumor xenografts, as well as advanced survivability under starvation serum conditions compared with the parental cell line [18, 19]. As EGFRvIII is often co-expressed with EGFRwt [20], both represent viable therapeutic targets for patients with GBM.

Sym004 is a 1:1 mixture of two recombinant human-mouse chimeric mAbs (futuximab and modotuximab) directed against non-overlapping EGFR epitopes. Preclinical studies with Sym004 have demonstrated activity against a variety of EGFR-expressing solid tumors [21–24]. Additionally, Sym004 has shown superior tumor growth inhibition in a range of xenograft models compared with other monoclonal anti-EGFR antibodies [21–24]. Sym004 is currently being evaluated in mCRC and GBM and has shown promising results in a phase I metastatic colorectal cancer trial with patients with chemotherapy-resistant/refractory tumors and acquired resistance to cetuximab and/or panitumumab [25]. The aim of the present study was to characterize the efficacy of Sym004 against a panel of EGFR positive patient-derived GBM xenografts with and without concomitant EGFRvIII expression.

## Materials and methods

All patient-derived xenograft models used in this study were obtained from the Duke Brain Tumor Biorespository after receiving appropriate written consent by the Duke University Institutional Review Board. The final diagnosis of tumor samples was made and confirmed by the Duke Pathology Department. All models used have been cryopreserved and have extensive snap and viably frozen aliquots for testing and thawing. In general, all xenograft lines have been profiled with short tandem repeat using ABI Profiler and CO*filer* commercial reagent kits made by Applied Biosystems. These kits are used for DNA typing of all xenograft lines.

### Selection of xenografts

**Fig. 1:**
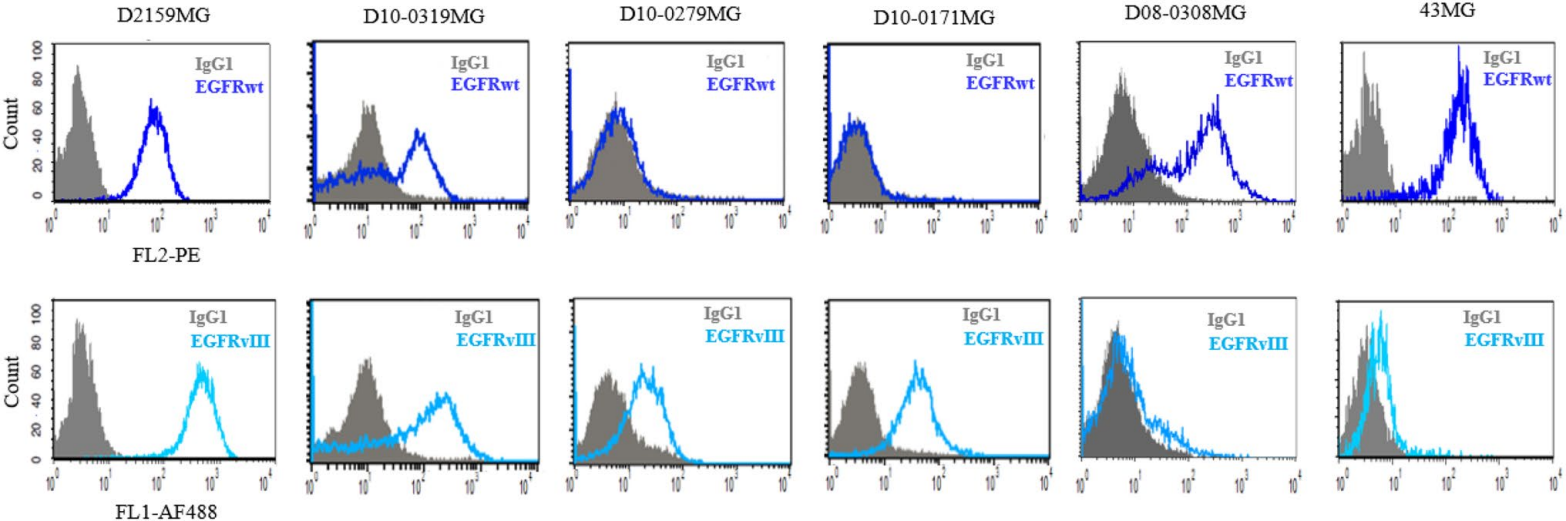
EGFR Expression on GBM Xenograft Cells by QFACS. The number of EGFRwt and EGFRvIII receptors expressed on cells isolated from xenografts was determined by QFACS. EGFRwt expression is featured in the upper panel and EGFRvIII expression in the lower panel. A shift to the right in the panels indicates presence of EGFRwt and/or EGFRvIII in xenografts used in this study

EGFRwt and EGFRvIII statuses were determined by quantitative fluorescent activated cell sorter (QFACS) analysis. D08-0308MG and 43MG were found to express EGFRwt only. D10-0279MG and D10-0171MG were found to express only EGFRvIII, whereas D10-0319MG and D2159MG expressed both EGFRwt and EGFRvIII (Fig. 1). Xenograft models D270MG and D317MG were previously described in literature and not retested for this study, both co-express EGFRwt and EGFRvIII [26–28].

### Dissociation of xenografts

Tissue from human biopsy-derived malignant glioma xenografts was obtained under sterile conditions from the Cancer Center Isolation Facility at Duke. Tumor tissue was processed and prepared for cell culture in a laminar flow hood under sterile conditions. The tumor material was finely minced and digested with 100 μg of Liberase (Roche Indianapolis, IN). This mixture was stirred at 37 °C for 10 min, and a cell-rich supernatant was obtained. The cells were washed with a complete zinc option (ZO) medium, further treated with Ficoll-Hypaque to remove any red blood cells, and then washed once more in a ZO medium as described in previous studies [29, 30].

### Determination of receptor number by quantitative FACS analysis

The number of EGFRwt and EGFRvIII receptors expressed on cells isolated from xenografts was determined by QFACS, using the Quantum Simply Cellular anti-Mouse IgG kit (Bangs Laboratories, Inc., Fishers, IN), as described in previous studies [29, 30]. To summarize, a cocktail of uniform-sized beads (one blank and four with varying capacities to bind to Mouse IgG) was used for creating a standard curve and the cells were stained with 10 µg/mL of Mouse IgG2b-PE, EGFR1-PE (an EGFRwt-specific antibody), Mouse IgG1-AF488, or L8A4-AF488 (an EGFRvIII-specific antibody) for 45 min at 4 °C. After washing, the beads and the cells were analyzed on a Becton Dickinson FACSCalibur instrument. Analysis of receptor density was performed by interpolation with the bead standard curves using QuickCal analysis software provided with the kit. The QFACS assays were performed on at least two different occasions on all brain tumor cells.

### Animals

Male and female athymic mice (nu/nu genotype, Balb/c background, 6–8 weeks old) were used for all antitumor studies. The animals were maintained in an Allentown JAG75 PNC ventilated cage and rack system (Allentown, PA). All animal procedures conformed to the Institutional Animal Care and Use Committee’s and the National Institute of Health’s guidelines.

### Tumor xenografts and implantation

Patient-derived human GBM xenografts maintained at The Preston Robert Tisch Brain Tumor Center were used for all studies.

In preparation for subcutaneous (s.c.) transplantation, s.c. xenografts passaged in athymic mice were excised from the host mice under sterile conditions in a laminar flow containment hood and placed into a modified tissue press. The resulting homogenate was then loaded into a repeating Hamilton syringe dispenser. The tumor homogenate was injected s.c. into the right flank of the athymic mouse at an inoculation volume of 50 µL with a 19-gauge needle [31, 32].

For intracranial (i.c.) studies, s.c. xenografts passaged in athymic mice were excised from the host mice under sterile conditions in a laminar flow containment hood. The xenograft was minced and the cells were separated with a 60-mesh tissue cytosieve (BioWhittaker Inc., Walkersville, MD) into a ZO solution (Sigma-Aldrich, Allentown, PA), allowing for passage through a 25-gauge needle. After centrifugation, the supernatant was removed, and the cells were mixed 1:1 with methylcellulose. This mixture was then loaded into a repeating 250-/J Hamilton syringe (Hamilton, Co., Reno, NV) dispenser and injected i.c. at an inoculation volume of 10 µl. The i.c. injections were performed by placing a mouse into a stereotactic frame. A 1/2” midline skin incision was made. The bregma was located and the coordinates (2 mm lateral) were determined. A mounting holder on the frame supported the syringe containing the cells. A sterile 25-gauge needle attached to the syringe was introduced through the calvaria and into the brain at a depth of 4 mm. The needle was then pulled back 0.5 mm to create a well for the homogenate. The xenograft homogenate was injected and after 1 min the syringe was pulled up and a small amount of bone wax was placed to occlude the hole. The mouse was removed from the frame and wound clips were used to close the skin [31, 32]. Lidocaine and Bupivicaine were used to control pain.

### Subcutaneous tumor measurement

Subcutaneous tumors were measured twice weekly with hand-held vernier calipers (Scientific Products, McGraw, IL). Tumor volumes, V, were calculated with the following formula: ${\text{V}}({\text{m}}{{\text{m}}^3})=[{({\text{width}})^2} \times ({\text{length}})]/2$.

### Subcutaneous and intracranial xenograft therapy

For the s.c. tumor studies, groups of 7–10 mice were stratified by tumor volume and were treated when the median tumor volumes were an average of 200 mm^3^. For i.c. tumor studies, groups of mice were randomized 3 days after i.c. tumor implantation, as previously described [33]. Groups of tumor-bearing animals received either Sym004, cetuximab, or TMZ and were compared to untreated controls.

### Antibodies and dosing

Sym004, futuximab, and modotuximab were provided by Symphogen, Denmark. TMZ was purchased from Selleckchem (Houston, TX) and cetuximab was purchased from the Duke Inpatient Pharmacy. Sym004, futuximab, modotuximab and cetuximab were administered at a dose of 50 mg/kg intraperitoneally (i.p.) twice weekly for 5 weeks (10 treatments total) and TMZ at a dose of 50 mg/kg i.p. for 5 consecutive days.

### Evaluation of subcutaneous xenograft response

The response of the s.c. xenografts to treatment was assessed by a delay in tumor growth and differences in median values. Growth delay, expressed as T–C, was defined as the difference in days between the median time required for the tumors in the treated (T) and control (C) animals to reach a volume five times greater than that measured at the start of the treatment and/or reach a minimum volume of > 1000 mm^3^. Statistical analysis was performed using a SAS statistical analysis program. The Wilcoxon rank order test and Student t test were used [31, 34–38].

### Evaluation of intracranial xenograft response

The response of the i.c. xenografts to treatment was assessed by the percentage of increase in time to a specific neurologic endpoint (i.e., seizure activity, repetitive circling, 15% decrease in weight or decrease in appetite) or to moribund status. Statistical analysis was performed using the Wilcoxon rank order test, as previously described [31, 32, 37–39]. All animals were observed twice daily for signs of distress or development of neurological symptoms, at which time they were removed from the study and euthanized.

### Receptor internalization

After 1 day in medium with 2% serum, NR6M cells were incubated with 20 µg/mL Sym004, futuximab, modotuximab, cetuximab or the negative control mAb for the indicated periods. After treatment, cells were fixed, permeabilized, and incubated with anti-EGFR primary antibody (20ES04, Sheep, Fitzgerald). Subsequently, the nuclei, the primary antibody and treatment antibodies were stained with Hoechst, donkey anti-sheep IgG coupled to Alexa fluorophore 488 and goat anti-human IgG coupled to Alexa fluorophore 647, respectively. Images were acquired using Opera High Content Screening System with a 40× objective (PerkinElmer).

### Analysis of receptor modulation

Lysates of cells treated with 20 µg/mL mAbs or Sym004 for 24 or 48 h were prepared as described elsewhere [40]. Samples for Simple Western analysis of total receptor levels were processed under standard conditions in a Sally Simple Western instrument (ProteinSimple). Rabbit primary antibodies against EGFRvIII from cell signaling technology (all diluted 1:50) were used. Statistically significant differences between untreated and treatment groups were calculated by one-way ANOVA with Bonferroni test post hoc correction.

## Results

### Subcutaneous in vivo study

**Fig. 2:**
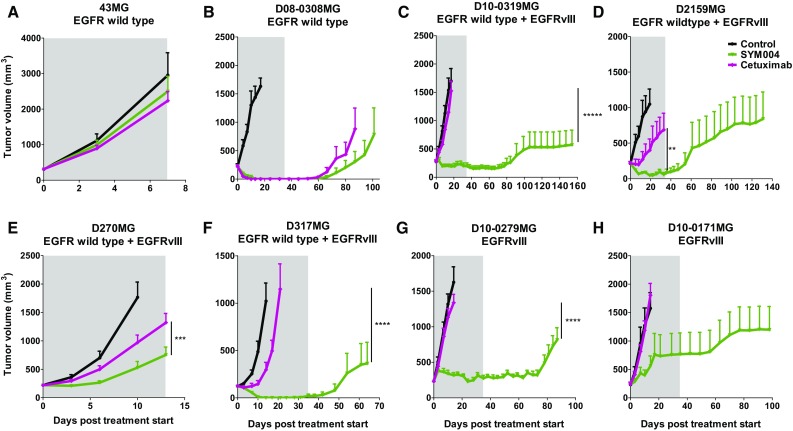
Effect of Sym004 in Subcutaneous Glioblastoma Xenograft Tumor Models. Tumor growth curves for subcutanous GBM xenografts expressing (**a, b**) EGFRwt, (**c**–**f**) EGFRwt + EGFRvIII, or (**g, h**) EGFRvIII. Homogenate of eight patient-derived GBM xenografts passaged in athymic mice were injected s.c. in the right flank of Balb/c nu/nu mice. At an average tumor size of 200 mm^3^, mice were randomized into four groups (N = 7–10/group) and treatment initiated. Sym004 and cetuximab were administered at a dose of 50 mg/kg i.p. twice weekly for 5 weeks (10 treatments in total). The grey area denotes the treatment period. Control treated mice are shown in black, Sym004 in green and cetuximab in mauve. Two-way ANOVA with Bonferroni’s multiple comparisons test was applied to compare tumor volumes at each time-point between Sym004 and cetuximab. Statistical analyses were performed using GraphPad Prism version 5.0 (GraphPad Software, Inc.). Data are presented as means ± SEM. *p < 0.05, ***p < 0.001, ****p < 0.0001

Sym004 produced statistically significant (P < 0.002) growth delays in seven of the eight subcutaneous GBM xenograft models tested (Fig. 2; Supplemental Table 1). The significant T–C values for the s.c. studies ranged from 7.80 to 91 days and Sym004 outperformed cetuximab in the 2 EGFRvIII-expressing models D10-0171MG and D10-0279MG and in xenografts expressing both EGFRwt and EGFRvIII (D10-0319MG, D2159MG, D317MG, and D270MG). The activity of cetuximab and Sym004 in the 2 EGFRwt xenograft lines (43MG and D08-0308MG) was similar.

### Intracranial in vivo study

**Fig. 3:**
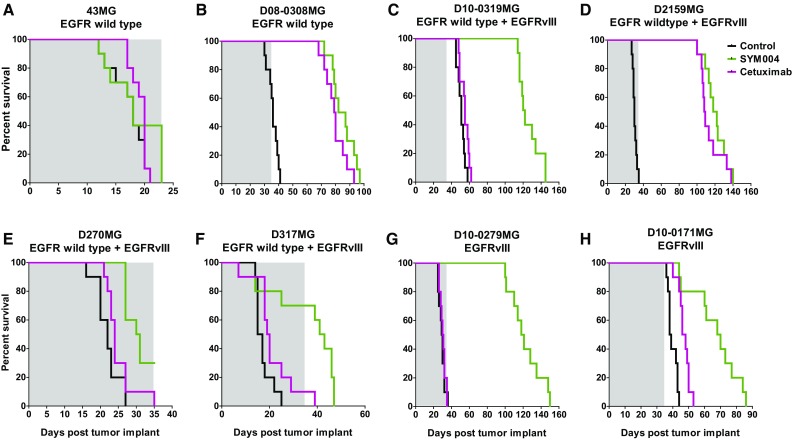
Effect of Sym004 in orthotopic glioblastoma xenograft tumor models. Kaplan–Meier survival curves for mice bearing intracranial GBM xenografts expressing (**a, b**) EGFRwt, (**c**–**f**) EGFRwt + EGFRvIII, or (**g, h**) EGFRvIII. Homogenate of eight patient-derived GBM xenografts passaged in athymic Balb/c nu/nu mice were injected i.c. using a stereotactic frame. Sym004 and cetuximab were administered at a dose of 50 mg/kg i.p. twice weekly for 5 weeks (10 treatments in total, N = 8–10/group). The grey area denotes the treatment period. Control treated mice are shown in black, Sym004 in green and cetuximab in mauve

The increase in percentage survival for the Sym004 i.c. studies ranged from 39 to 305% and Sym004 significantly increased survival in seven of the eight models tested (Fig. 3; Supplemental Table 2). In the intracranial studies, Sym004 statistically outperformed cetuximab in the EGFRvIII alone expressing xenografts (D10-0171MG and D10-0279MG). In the models expressing both EGFRwt and EGFRvIII, Sym004 significantly outperformed cetuximab in D270MG, D-317MG, and D10-0319MG but not in D2159MG. As seen in the s.c. models, the activity of cetuximab and Sym004 in the two EGFRwt xenograft lines (43MG and D08-0308MG) was similar.

### Internalization and degradation of EGFRvIII by Sym004

**Fig. 4:**
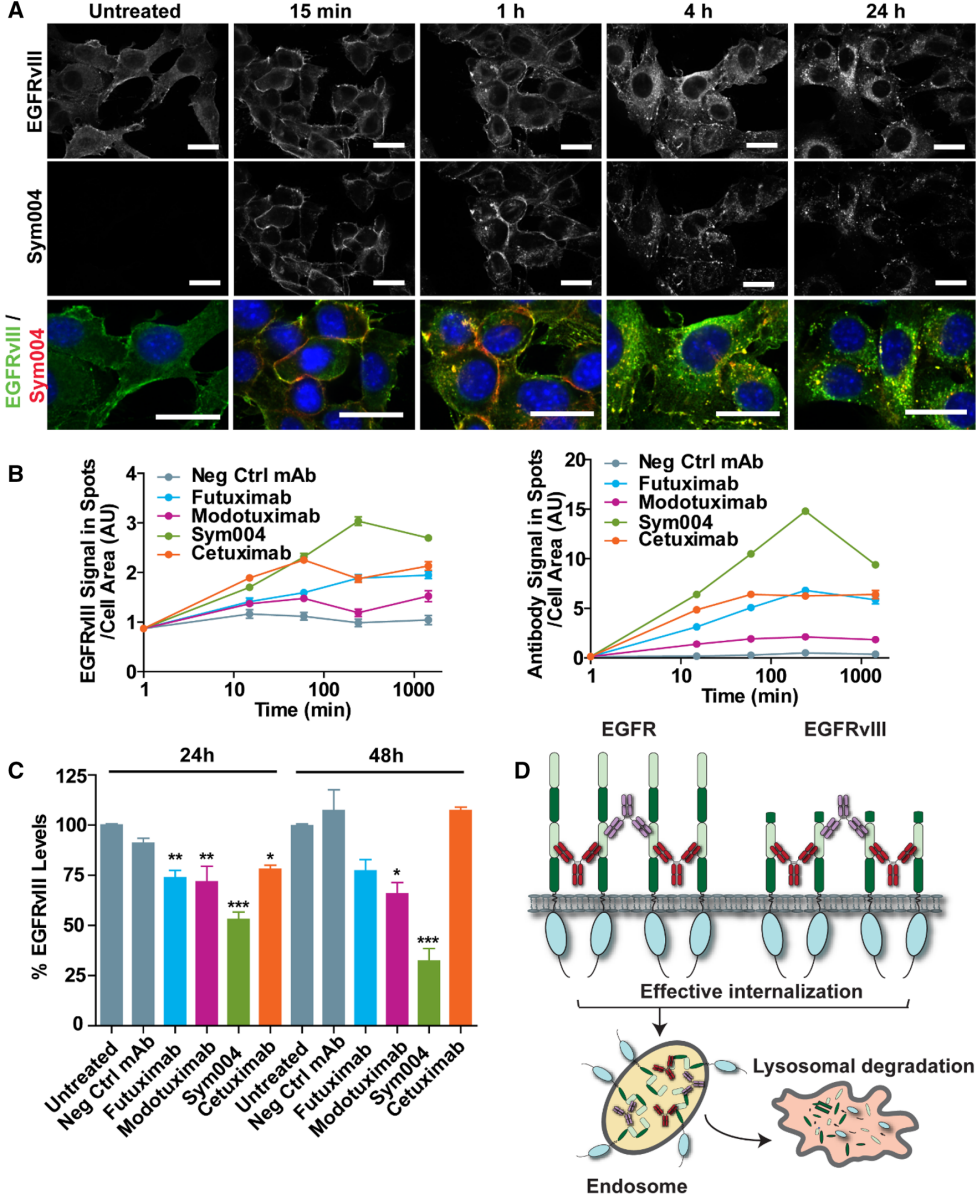
Sym004 Internalizes and Degrades EGFRvIII. **a** NR6M cells were incubated with Sym004 for the indicated periods, and localization of EGFRvIII and Sym004 was detected with fluorophore-coupled secondary antibodies. The upper two panels show localization of EGFRvIII and Sym004, respectively. The lower panels show magnified composite images. Green, red, and blue indicate localization of EGFRvIII, Sym004, and nuclei, respectively. **b** Quantification of EGFRvIII and antibodies detected in spots after antibody treatment for the indicated periods. **c** The cell line NR6M was treated with negative control mAb (Neg Ctrl mAb), futuximab, modotuximab, or Sym004 for 24 or 48 h, followed by harvest of cell lysates and detection of EGFRvIII by Western blot. Data are represented as means ± SEM. Statistically significant differences from untreated controls are indicated by asterisks *p < 0.05, **p < 0.01, ***p < 0.001. **d** Proposed model of internalization and degradation of EGFR and EGFRvIII by Sym004

The ability of Sym004 to induce EGFRvIII internalization and degradation was investigated in the EGFRvIII-expressing cell line NR6M. NR6M cells were incubated with Sym004, and the localization of EGFRvIII and Sym004 was detected with fluorophore-coupled secondary antibodies. Sym004 induced higher levels of EGFR internalization compared to individual antibodies (futuximab and modotuximab) and cetuximab (Fig. 4a, b) [41]. The fate of internalized receptors was investigated using quantitative Western blot analysis by Protein Simple. As shown in Fig. 4c, Sym004-treated NR6M cells had significantly lower levels of EGFRvIII compared to mAb-treated cells. After 48 h, Sym004 decreased EGFRvIII levels to approximately one-quarter of the levels in untreated cells, whereas mAbs had no impact or only slightly reduced EGFRvIII levels.

### Individual antibodies tested subcutaneously in vivo

**Fig. 5:**
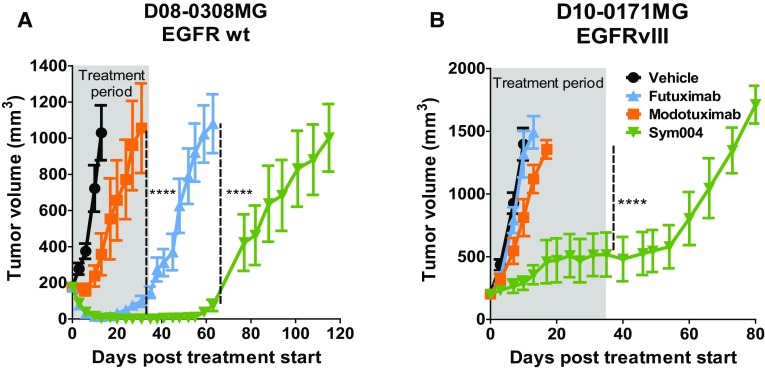
Effect of Sym004, and Its Components Futuximab and Modotuximab, in Subcutaneous Glioblastoma Xenograft Tumor Models. Homogenate of **a** EGFRwt (D08-0308MG) and **b** EGFRvIII (D10-0171MG) patient-derived GBM xenografts passaged in athymic mice were injected s.c. in the right flank of Balb/c nu/nu mice. At an average tumor size of 200 mm^3^, mice were randomized into four groups (N = 10/group) and treatment initiated. Sym004, futuximab, and modotuximab were administered at a dose of 50 mg/kg i.p. twice weekly for 5 weeks (10 treatments in total). The grey area denotes the treatment period. Control treated mice are shown in black, Sym004 in green, futuximab in blue, and modotuximab in orange. Two-way ANOVA with Bonferroni’s multiple comparisons test was applied to compare tumor volumes at each time-point between Sym004 and futuximab and modotuximab respectively. Statistical analyses were performed using GraphPad Prism version 5.0 (GraphPad Software, Inc.). Data are presented as means ± SEM. *p < 0.05, ***p < 0.001, ****p < 0.0001

To test the hypothesis that the individual mAbs were working synergistically as an inhibitor of tumor growth, individual antibodies were tested in two s.c. models using D08-0308MG (EGFRwt) and D10-0171MG (EGFRvIII) and then compared to Sym004 (Fig. 5). In both the EGFRwt and EGFRvIII expressing xenograft models, Sym004 statistically outperformed the control and each individual antibody.

## Discussion/Conclusion

GBM is the most common brain cancer in adults. Despite considerable attention from the research community, it remains a major therapeutic challenge with extremely poor clinical outcomes. Overexpression and amplification of EGFR, the most common genetic alternation in primary GBM, are associated with more aggressive and drug-resistant tumor phenotypes [42]. Of the primary GBMs that overexpress EGFR, up to 60% also express the tumor-specific variant, EGFRvIII [43]. Expression of EGFRvIII has been shown to lead to significant tumor and cell growth advantage in vivo and in vitro settings [18, 19]. These pro-tumorigenic properties of EGFRvIII can be linked to resistance to conventional therapies [13]. Unfortunately, most trials to date targeting EGFR and/or EGFRvIII have demonstrated little to no benefit [20, 44, 45]. Several clinical trials have evaluated the anti-EGFR mAb cetuximab. Though well tolerated by patients alone and in combination with other agents, only a small percentage of patients had an improvement in overall survival and experienced durable disease control. These studies indicate that patients with EGFR amplification had better responses than those without. Despite these encouraging results, there exists clear opportunities for continued development regarding therapies targeting the EGFR. Thus, the development of more efficacious cancer treatments and therapeutic options is extremely important for effective treatment in order to extend life and/or cure patients with GBM [46, 47].

The results from this study clearly demonstrate that Sym004 is active against patient-derived GBM xenografts expressing EGFR and/or EGFRvIII. In addition, it was shown that the two mAbs, futuximab, and modotuximab, comprising Sym004 work additively and even synergistically in the tested GBM models. The superior tumor growth inhibition induced by Sym004 in several of the s.c. GBM models and the prolonged survival upon Sym004 treatment in the i.c. models compared to cetuximab, demonstrate the potential of Sym004 for treatment of GBM.

EGFRvIII is constitutively active, unable to bind EGFR ligands and escapes downregulation due to inefficient internalization and/or transport to lysosomes. A potential explanation for the superior activity of Sym004 compared to the individual mAbs and cetuximab could be the ability of Sym004 to induce EGFR, cross-linking, internalization and degradation [22].

The two antibodies futuximab and modotuximab bind non-overlapping epitopes on domain III of EGFR, a domain that is intact in EGFRvIII. It was therefore expected that Sym004 were able to induce internalization and degradation of EGFRvIII similar to EGFRwt. Indeed, we were able to demonstrate that Sym004 induces efficient internalization and degradation of EGFRvIII thereby effectively shutting down oncogenic signaling. Sym004 also engages secondary effector functions such as antibody dependent cellular cytoxicity (ADCC) and/or complement dependent cytoxicity (CDC), which contributes to the drug candidates overall anti-tumor activity [21].

Sym004 thus has the unique potential to effectively target both EGFRwt and EGFRvIII in GBM providing a clear rationale for evaluating Sym004 in patients with GBM. An ongoing phase 2 trial in patients with GBM will assess the safety and efficacy of Sym004 in patients with progression after primary therapy.

## Electronic supplementary material

Below is the link to the electronic supplementary material.

Supplementary material 1 (DOCX 26 KB)

Supplementary material 2 (DOCX 27 KB)
