# Supplementary material for: Sym004-induced EGFR elimination is associated with profound anti-tumor activity in EGFRvIII patient-derived glioblastoma models
Source: J Neurooncol. 2018 Mar 21;138(3):489–98. doi: 10.1007/s11060-018-2832-6 (PMC5999169; doi:10.1007/s11060-018-2832-6)
Supplement: Supplementary file 1 — Supplementary material 1 (DOCX 26 KB) [file 11060_2018_2832_MOESM1_ESM.docx]

**Supplemental Table 1**

|  |  | Subcutaneous Model | | | | | |
| --- | --- | --- | --- | --- | --- | --- | --- |
| Sym004 vs Cetuximab & TMZ | | Sym004 | | Cetuximab | | TMZ | |
| Xenograft Line | EGFR Status | T-C (days) | P Value | T-C (days) | P Value | T-C (days) | P Value |
|  |  |  |  |  |  |  |  |
| 43MG | wildtype | 0.11 | 0.302 | 0.57 | 0.170 | 0.56 | 0.432 |
| D08-0308MG | wildtype | 86 | 0.002 | 80 | 0.002 | 73 | 0.001 |
| D270MG | vIII | 7.80 | 0.001 | 3.20 | 0.035 | n/e | n/e |
| D317MG | vIII | 72 | 0.001 | 4.60 | 0.032 | n/e | n/e |
| D2159MG | vIII | 91 | 0.001 | 71 | 0.005 | 92 | 0.005 |
| D10-0171MG | vIII | 53 | 0.001 | 0.33 | 0.398 | 45 | 0.001 |
| D10-0279MG | vIII | 78 | 0.001 | 1.77 | 0.176 | 106+ | 0.001 |
| D10-0319MG | vIII | 38 | 0.001 | 5.00 | 0.016 | 114 | 0.001 |
| *n/e - not evaluated | |  |  |  |  |  |  |
