# Supplementary material for: Sym004-induced EGFR elimination is associated with profound anti-tumor activity in EGFRvIII patient-derived glioblastoma models
Source: J Neurooncol. 2018 Mar 21;138(3):489–98. doi: 10.1007/s11060-018-2832-6 (PMC5999169; doi:10.1007/s11060-018-2832-6)
Supplement: Supplementary file 2 — Supplementary material 2 (DOCX 27 KB) [file 11060_2018_2832_MOESM2_ESM.docx]

**Supplemental Table 2**

|  |  | Intracranial Model | | | | | |
| --- | --- | --- | --- | --- | --- | --- | --- |
| Sym004 vs Cetuximab & TMZ | | Sym004 | | Cetuximab | | TMZ | |
| Xenograft Line | EGFR Status | % Increase | P Value | % Increase | P Value | % Increase in Survival | P Value |
|  |  | in Survival |  | in Survival |  |  |  |
| 43MG | wildtype | 0% | 0.315 | 11% | 0.140 | 14% | 0.095 |
| D08-0308MG | wildtype | 135% | 0.001 | 121% | 0.001 | 76% | 0.001 |
| D270MG | vIII | 39% | 0.001 | 9% | 0.023 | n/e | n/e |
| D317MG | vIII | 163% | 0.001 | 26% | 0.013 | n/e | n/e |
| D2159MG | vIII | 303% | 0.001 | 261% | 0.001 | 260% | 0.001 |
| D10-0171MG | vIII | 79% | 0.001 | 22% | 0.001 | 83% | 0.001 |
| D10-0279MG | vIII | 305% | 0.001 | 3% | 0.256 | 400% | 0.001 |
| D10-0319MG | vIII | 137% | 0.001 | 8% | 0.038 | 143% | 0.001 |
| *n/e - not evaluated | |  |  |  |  |  |  |
